# Sudden Cardiac Death in Children Affected by Cardiomyopathies: An Update on Risk Factors and Indications at Transvenous or Subcutaneous Implantable Defibrillators

**DOI:** 10.3389/fped.2020.00139

**Published:** 2020-04-03

**Authors:** Valeria Rella, Gianfranco Parati, Lia Crotti

**Affiliations:** ^1^Istituto Auxologico Italiano, IRCCS, Department of Cardiovascular, Neural and Metabolic Sciences, San Luca Hospital, Milan, Italy; ^2^Department of Medicine and Surgery, University of Milano-Bicocca, Milan, Italy; ^3^Istituto Auxologico Italiano, IRCCS, Center for Cardiac Arrhythmias of Genetic Origin, Milan, Italy; ^4^Istituto Auxologico Italiano, IRCCS, Laboratory of Cardiovascular Genetics, Milan, Italy

**Keywords:** ICD (implantable cardioverter-defibrillator), dilated cardiomyopathy, Arrhythmogenic cardiomyopathy, cardiomyopaphy, hypertrophic, sudden cardiac death (SCD)

## Abstract

In the present paper, we will discuss the main cardiomyopathies affecting children with a specific focus on risk stratification and prevention of sudden cardiac death (SCD). We will discuss the main clinical features of hypertrophic cardiomyopathy (HCM), dilated and restrictive cardiomyopathies, left ventricular non-compaction (LVNC) and arrhythmogenic cardiomyopathy (AC), always highlighting their peculiarities in the pediatric age. Since sudden cardiac death may be the first manifestation of the disease, even in children, the identification of the specific underlying condition and of risk factors are pivotal to carry out the appropriate preventing strategies. ICD recommendations in children are similar to adults, but supporting evidences are not so solid, being based on registries or single center studies. Furthermore, children and young patients are most likely to manifest long term complications related to an implanted ICD, and this should be taken into account when evaluating the risk benefit ratio. In this perspective, subcutaneous ICDs (S-ICDs) could carry an advantage; however, they cannot be considered in small children for technical reasons. Data on effectiveness and safety of S-ICDs in a pediatric population is still lacking, although some limited experiences are reported and will be discussed in the current review.

## Introduction

Pediatric cardiomyopathies (CMP) are rare with an annual incidence of 1,1-1,5/100.000 in children below age 18 (1, 2). Affected adult and children may have similar clinical presentations, but their outcome may differ considerably. Data on pediatric cardiomyopathies are mainly based on large international registries and small single center studies (1–11). Consistent evidence about risk factors for sudden cardiac death (SCD) and preventing strategies along with disease specific therapies is still lacking, and larger trial would be desirable.

This review will address the main cardiomyopathies affecting children with a specific focus on risk stratification and prevention of sudden cardiac death (SCD).

## Pediatric Cardiomyopathies

Pediatric cardiomyopathies are usually classified according to phenotypic features (2) and in large registries, such as the North America Pediatric Cardiomyopathy Registry (PCMR, 1994) (12), dilated cardiomyopathy (DCM) and hypertrophic cardiomyopathy (HCM) are the most common conditions with annual incidence of 0,57 and 0,47/100.000 (1, 12). Other data show an even greater prevalence of HCM in children, with an estimation of 2.9/100.000 (1, 2, 11). By contrast, restrictive cardiomyopathy (RCM) is less frequently observed in pediatric age, while overlapping phenotypes of HCM/RCM are more often described (1, 2, 13). Non-compaction cardiomyopathy (NCCM) stays in the middle with an incidence varying from 0,12 to 0,81/100.000 according to age; although, several more cases were diagnosed in recent years suggesting a higher incidence (14). The same applies for arrhythmogenic cardiomyopathy (AC), that has been historically described as a condition affecting young-adults, but recently an increasing number of children diagnosed with this condition have been described (15).

Clinical evaluation and imaging could be more challenging in children, but the early identification of sign and symptoms suggestive for secondary forms of cardiomyopathies that could require specific treatments, is extremely important.

## Sudden Cardiac Death in Pediatric Cardiomyopathies

**Figure 1:**
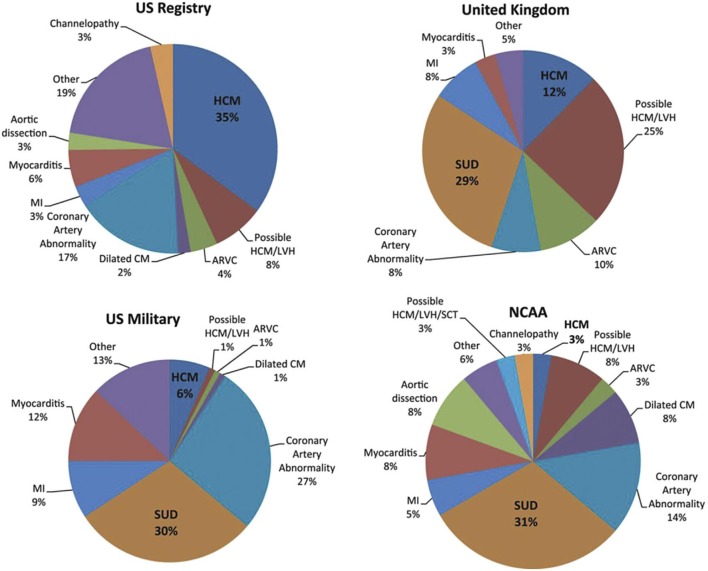
Causes of SCD are heterogeneous between studies. The major cause of SCD among cardiomyopathies is HCM, followed by AC and DCM. CM, cardiomyopathy; MI, myocardial infarction; NCAA, National Collegiate Athletic Association; SCT, sickle cell trait; SUD, sudden unexplained death [From Elizabeth et al. (23); with permission].

SCD in pediatric cardiomyopathies is a rare but devastating event with an incidence between 1,3 and 8,5/100.000 patient-years (16). An effective preventing strategy in this population is difficult mainly due to the heterogeneity of underlying causes. In the majority of children aged from 1 to 4 years old, SCD is due to electrical conditions. In contrast, structural heart diseases are more likely to be found in older children (17, 18). SCD incidence depends mostly on age, gender and phenotype. Regarding age and gender, the group from 5 to 10 years has a lower risk compared to the group aged 1–4 years and to the group older than 15 years. Furthermore, the risk is significantly higher among males than females with a 2:1 ratio (19). As far as phenotype is concerned, the major cause of SCD among cardiomyopathies is HCM, followed by AC and DCM (20–23) (Figure 1).

ICDs recommendations for pediatric patients mostly refer to adult guidelines (24, 25) even if supporting evidence is more limited. Indeed, risk factors identified in adults are used in children as well, despite the absence of an adequate validation. Therefore, ICD for secondary prevention is indicated for survivors of cardiac arrest, hemodynamically unstable ventricular tachycardia (VT) or sustained stable VT (26). While, for primary prevention, indications are disease-specific (Table 1). As an example, in dilated cardiomyopathy an ICD is recommended in children with severely reduced LVEF (30–35%) (36) and in those awaiting heart transplant (37).

**Table 1:** A summary of the main risk factors for sudden cardiac death recognized for HCM, DCM, LVNC, and AC in the pediatric population.

**Guidelines and Studies**	**CMP**	**Risk factors for sudden cardiac death**
ESC Guidelines (24)	HCM	Maximum LV wall thickness >30 or z-score >6, unexplained syncope, NSVT, and family history of SCD
AHA-ACC Guidelines (25)	HCM	Major: family history of sudden cardiac death, extreme left ventricular hypertrophy, unexplained syncope; Minor: non-sustained VT, hypotensive blood pressure response to exercise
Maron et al. (27)	HCM	Major: family history of sudden cardiac death, extreme left ventricular hypertrophy, unexplained syncope; Minor: non-sustained VT, hypotensive blood pressure response to exercise
Norrish et al. (28) Meta-analysis	HCM	Major: previous adverse cardiac event, non-sustained ventricular tachycardia, unexplained syncope and extreme left ventricular hypertrophy; Intermediate: left atrial diameter; Minor: age at presentation and gender, symptoms, ECG abnormalities, abnormal blood pressure response to exercise, LVOT obstruction and family history of SCD
Maurizi et al. (29)	HCM	Symptoms at onset Thin filament mutations
Norrish et al. (30)	HCM	Unexplained syncope, NSVT, LA diameter, degree of LVH
PCMR	DCM	Diagnosis at age <14.3 years, LV dilation, LV posterior wall thinning (<14 mm) at presentation, HF, low EF, antiarrhythmic drugs
ESC and American Guidelines	DCM	LVEF <35% in patients NYHA II-III, despite optimal medical therapy
NAACS	LVNC	Systolic dysfunction, arrhythmias
Te Riele et al. (31)	AC	Probands, 500 PVCs/24 h, structural abnormalities
De Witt et al. (15)	AC	Probands, RV disease, PKP2 variants, LV disease, cardiac inflammation
ESC Guidelines (24)	AC	NSVT, LVEF <45%, male sex, and non-missense mutations
AHA-ACC Guidelines (25)	AC	Resuscitated SCA, sustained VT, ventricular dysfunction with left or right ventricular EF <35%, syncope
Consensus document (32)	AC	Major: NSVT, inducibility to VT at EPS and LVEF ≤ 49%; Minor: male sex, >1,000 premature ventricular contractions/24 h, RV dysfunction, proband status, 2 or more desmosomal variants
Ferreiro-Marzal et al. (33), Lambiase et al. (34), and Protonotarios et al. (35)	AC	Phospholamban mutations, FLMC, and LMNA mutations

## Clinical Profile and Risk Factors for SCD

### Hypertrophic Cardiomyopathy

The etiology of HCM in children is heterogeneous including sarcomeric phenotypes and phenocopies such as the RASopathies, metabolic storage disorders, neurodegenerative and mitochondrial diseases (1, 3, 38). While sarcomeric phenotypes are commonly diagnosed during adolescence or early adulthood, the previously mentioned phenocopies have an earlier occurrence and are often associated with neurologic and muscular abnormalities (39). The long-term prognosis largely depends on the etiology. The overall rate of ventricular arrhythmias is higher in children compared to adults and the most common cause of mortality is SCD (39). Children with worse prognosis tend to have mixed phenotypes, heart failure, lower ejection fraction and higher posterior wall thickness or septal thickness at the time of diagnosis (40). Similar to adults, children should be advised to stop competitive sports (41, 42). On the other hand, it is not clear if beta-blockers significantly reduce the risk of sudden death, since there are only few data supporting their protective role (43). Another debated issue is the role of septal reduction in decreasing the risk of SCD. Indeed, even if there are some evidences pointing out that effective septal reduction may reduce SCD risk in adults, conclusive results are not yet available (44). Data are even more scarce in the pediatric population, in which, at variance to the adult population, the LVOT gradient is not consistently linked to SCD risk (45).

Regarding risk stratification and indication for ICD implant for primary prevention, European (24) and American guidelines (25) are not fully concordant. Indeed, European guidelines propose a risk model calculator (24) that however is not validated for children below 16 years. In children, European Guidelines recommend the use of four major risk factors: maximum LV wall thickness >30 or z-score >6, unexplained syncope, NSVT and family history of SCD and an ICD is indicated whenever 2 or more clinical risk factors are present (24). American guidelines suggest the same risk stratification scheme for children and adults affected by HCM. Specifically, five risk factors are considered; three are major risk factors [family history of sudden cardiac death, extreme left ventricular hypertrophy (LVH), unexplained syncope] and in the presence of one of them the ICD is recommended with a class IIa indication. The remaining two are minor risk factors (non-sustained VT, hypotensive blood pressure response to exercise), and in the presence of a single minor risk factor ICD may be considered but its benefit is uncertain (25). Maron et al. (27) supported this risk stratification strategy in 2013 in a population of 224 pediatric patients implanted with an ICD (188 patients were implanted in primary prevention, 77% had a sarcomeric HCM and syndromic cases were excluded). ICD discharge rates per year were quite high especially in the subset of patients implanted in secondary prevention (14%); in those in primary prevention ICD discharge rate was 3,1% per year and extreme LVH was the most common risk factor, followed by family history and unexplained syncope (27). Recently, a validation study of the ESC guidelines criteria has been published by Norrish et al. (30). The study enrolled 411 pediatric patients implanted with an ICD and followed for a median period of 5.5 years. Importantly, HCM phenocopies such as Rashopaties, metabolic disorders and neuromuscular diseases were excluded from the analysis. Among these patients, 280 patients were implanted with no risk factors, 113 with a single risk factor and only 16 with two or more risk factors. Therefore, the vast majority of the patients were implanted without an indication according to ESC guidelines (24). In this cohort, major arrhythmic events were higher for patients with increasing number of clinical risk factors, however the positive predictive value of treatment threshold was low, leading to unnecessary implants in many patients with consequent long-term complications (30).

Other studies worth of note recently published, all excluding phenocopies, have highlighted additional risk factors which may have a role in the risk stratification of children affected by HCM. In 2017 a systematic meta-analysis was conducted by Norrish et al. (28) on 3,394 patients below 18 years from 25 studies, and four major risk factors have been identified including previous adverse cardiac event, non-sustained ventricular tachycardia, unexplained syncope and extreme left ventricular hypertrophy. Along with these major criteria, left atrial diameter has been pointed out as an additional important risk factor (28). More recently, Norrish et al. (45) developed a novel risk prediction model for sudden cardiac death in HCM children through a retrospective, multicenter, longitudinal cohort study on 1,024 patients below 16 years and the most predictive variables included in the model were unexplained syncope, degree of hypertrophy, LA diameter and NSVT, while the maximal LVOT gradient appeared to be inversely related with SCD risk, a data that clearly needs further investigation. Finally, Maurizi et al. (29) described the long-term outcome of 100 pediatric patients with HCM, with a 40-year follow-up. Interestingly, genetic data emerged as major predictors of SCD at multivariate analysis. Indeed, patients with any thin filament mutations (i.e., Troponin I and T mutations) had an increased risk of cardiac events while neither extreme LVH nor NSVTs were associated with increased risk (29). In summary, a general consensus on SCD risk stratification for children affected by sarcomeric HCM is still not available; however, a growing number of publications are pointing to specific risk factors summarized in Table 1. Importantly, these factors have been validated for sarcomeric HCM, while HCM phenocopies, having a different etiology, bring different risk patterns specific for each disease.

### Restrictive Cardiomyopathy

Pediatric restrictive cardiomyopathies may have different presentations, which range from asymptomatic patients to heart failure, syncope or sudden death. Sarcomeric genes are the genes most commonly implicated. Sudden cardiac death may be caused by ventricular arrhythmia or heart block (46). The largest cohort published showed a 5-year-survival from diagnosis of 68% (13). Pure RCM has a worse prognosis than combined forms.

There are few factors that are recognized to influence the outcome such as congestive heart failure, tachyarrhythmias, ischemia, and pulmonary hypertension (13, 47, 48).

RCM has limited therapeutic solutions and no known risk factors for sudden cardiac death. Unfortunately, for some of these patients, heart transplantation is the only therapeutic option.

### Dilated Cardiomyopathy

The PCMR Registry, including 1,803 children with dilated cardiomyopathy, is one of the largest cohorts to date, showing that a previous myocarditis is the most frequent cause of DCM in children, accounting for 40% of the cases (49–51). Other recognized causes are toxic and genetic (52, 53). Data show that 20% of children diagnosed with DCM normalize their systolic function within 2 years; whereas 40% die or undergo heart transplantation (54–58). In patients with neuromuscular, metabolic or mitochondrial disorders the underlying disease is an important determinant of outcome (1, 3, 59).

Clinical presentation in children may vary from asymptomatic to heart failure and cardiogenic shock (60). Compared to adults, children with heart failure have greater morbidity and mortality (61). Medical therapy may be challenging due to lack of clinical trials specifically designed for children.

SCD is a rare event in children with DCM; ventricular arrhythmias and cardiac fibrosis are less prevalent than in adults and mortality predominantly occurs from pump failure (62). Risk factors for SCD identified in children with DCM are: early age at diagnosis, LV dilatation, LV posterior wall thinning (<14 mm) at presentation, heart failure, low ejection fraction (55, 63, 64). However, there are no specific recommendations for ICD implant in the pediatric population according to guidelines, and therefore adult criteria should be applied. Specifically, European guidelines suggest an ICD in the presence of hemodynamically no tolerated VT or VF (class Ia) and in primary prevention in patients symptomatic for heart failure (NYHA II-III), with ejection fraction (EF) <35% despite optimal medical therapy (class Ib) (24). Similarly, American guidelines indicate an ICD for primary prevention in patients with symptoms of heart failure and EF <35% despite optical medical therapy even though with a class Ia indication (25).

### Left Ventricular Non-compaction

The incidence of LVNC is estimated to be 0,12/100000 in children below age 10, with a peak of 0,81/100000 in the first year of life (14).

The presentation may occur as isolated phenotype (23%), mixed HCM/LVNC (11%), or mixed LVNC/DCM (59%) (65). The clinical phenotype in children ranges from a benign course to a severe progressive systolic or diastolic dysfunction, life-threatening arrhythmias, or thromboembolism.

Thromboembolic disease occurs in up to 24% of adults (66). No data have been reported regarding the risk or occurrence of thromboembolic disease in pediatric patients. Children with a dilated or mixed phenotype tend to have a worse prognosis. On the contrary, normal systolic function has been associated to better outcome (66). Similar to DCM, systolic dysfunction and arrhythmias are independent risk factors for sudden death and children presenting with these phenotypes should be considered for an ICD (67).

Recently, calmodulin mutations have been associated with few cases in which the long QT Syndrome phenotype was overlapping with LVNC (68). These patients, are characterized by life-threatening arrhythmias occurring very early in life and typically induced by adrenergic activation. Available pharmacological therapies are usually insufficient and ICD is probably necessary in most of these cases (68).

### Arrhythmogenic Cardiomyopathy

Diagnostic criteria in AC are tailored for adult patients; indeed, some of the features, as early precordial T-wave inversion, may represent a normal finding in children and other aspects, as Epsilon waves or regional dysfunction may be more difficult to be detected (69). Furthermore, some testing modalities in pediatric patients are more difficult; indeed, echocardiography is more easily performed after age 3 years and cardiac MRI, without anesthesia, after age 8 years, as highlighted by a recently published European consensus document (32). There are two rare forms of AC which are peculiar of the pediatric age: the Carvajal syndrome (ARVC8) and the Naxos disease (ARVC12), both transmitted as autosomal recessive disorders. The Naxos disease, due to plakoglobin gene mutations, is characterized by a severe cardiac phenotype, with 100% penetrance by adolescence and an annual rate of SCD of 2.3%, associated with wooly hair and palmoplantar keratoderma (35). Similarly, homozygous, or compound heterozygous mutations in the desmoplakin gene determine a variant of the Naxos disease which is known as Carvajal syndrome, characterized by the same cutaneous phenotype and a predominantly left ventricular involvement, overlapping with dilated cardiomyopathy (70).

Regarding the more common autosomal dominant form of AC, there are very few studies specifically focused on pediatric patients. One of the largest cohorts, including 75 patients below age 18, is the one by te Riele et al. (31), showing that AC with a pediatric onset was characterized by a high proportion of sudden cardiac death or arrest at presentation. Once diagnosed, outcomes such as sustained VT, cardiac transplantation and death were similar to adult patients. Children with adverse events were more likely to be probands, to have >500 PVCs/24 h and to have structural abnormalities on imaging. In this cohort of pediatric patients fulfilling the 2010 Task Force diagnostic criteria (31), males were more frequently represented, with a mutation in plakophilin-2 (76%) and they were often involved in endurance sport, thus highlighting that participation in high-intensity activity may influence an early onset of the disease. Indeed, both European, and American guidelines recommend avoidance of competitive sports in affected individuals as class I indication, as intense physical activity favors progression of the disease and sudden cardiac death risk (24, 25).

The most recent cohort of pediatric AC patients published is the one by De Witt et al. (15) which included 32 cases with predominant right ventricular (*n* = 16), left dominant (*n* = 7) or biventricular (*n* = 9) AC. Cardiac arrest and sustained VT tended to occur more frequently in probands with the predominant right form of the disease, in which PKP2 variants were more often identified (15).

Despite these few data, there are no specific guidelines, regarding ICD recommendation for the pediatric population and the indications are applied following adult guidelines. European Guidelines (24) recommend an ICD for secondary prevention in case of aborted SCD and not tolerated VT (class I) or for well-tolerated sustained VT balancing the risk of long-term complications (class II). As far as primary prevention is concerned, an ICD is indicated in case one or more risk factors are recognized (24), namely unexplained syncope, frequent NSVT, family history of premature SCD, extensive RV and LV dysfunction, and VT induction on EP study.

On the other hand, American guidelines strongly suggest an ICD in the presence of one risk marker among resuscitated SCA, sustained VT, ventricular dysfunction with left or right ventricular EF <35% (class I) or syncope of suspected arrhythmic origin (class II) (25). Recently, a collaboration of 18 centers from North America and Europe developed a risk model (71) which provided a 5 year prediction of SCD. However, most of the patients included in the study were Caucasian with pathogenic variants primarily identified in the plakophilin-2 gene; therefore, it is too premature to extrapolate these results and to use such a risk model to other ethnic background or genotypes (71).

**Figure 2:**
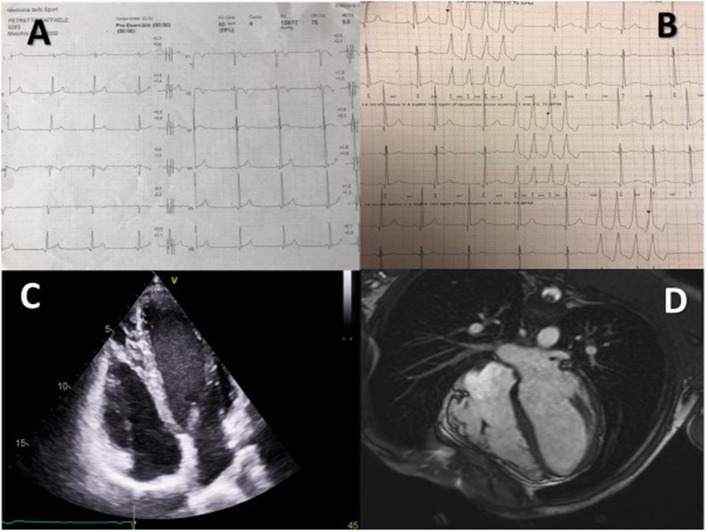
A 15 year-old male proband affected by AC, considered for an S-ICD. **Table A** shows typical T wave inversion in V1–V2 and diphasic T wave in V3, B shows recurrent NSVTs, C shows structural alterations in the right ventricle and D shows dysmorphic and diskinetic aspects of the right ventricle in the cardiac MRI.

All these specific risk factors for SCD in AC have extensively been re-discussed in the more recent Consensus Document for AC in 2019 and once again no specific recommendations are provided for the pediatric age (72). According to the authors, in case of primary prevention, an ICD should be evaluated by means of a risk score based on the presence of major or minor risk markers. In details, major criteria are represented by NSVT, inducibility to VT at EPS and LVEF ≤ 49%. Minor criteria include male sex, >1,000 premature ventricular contractions/24 h, RV dysfunction, proband status, 2 or more desmosomal variants (72) (Figure 2).

An additional specific risk stratification is suggested according to the genotype. In this perspective, in patients with AC and phospholamban mutations associated with LVEF <45% or NSVT, an ICD is reasonable (73), this being the case also in patients with FLNC AC and LVEF <45% (35) or Lamin A/C mutations and two or more of the following: LVEF <45%, NSVT, male sex, non-missense mutations (74).

## ICD for Prevention of SCD

When a disease at risk for sudden cardiac death is identified in a young person, ICD implantation based on risk stratification is pivotal.

The largest group of children with ICDs are those with primary electrical disease (40%), followed by cardiomyopathies and congenital heart disease (30% each) (75, 76).

The efficacy of ICD in children has been demonstrated in several studies with a rate of appropriate shocks ranging from 11% (30) to 20% (27) per year, with a prevalence of appropriate therapy in those implanted for secondary vs. primary prevention (27, 76). However, the benefits of ICD therapy might be overcome by long-term complications, especially in young patients. Complications in transvenous-ICD (T-ICD) range between 3 and 8% in adult population (77). These events may be more relevant in the young because of a long exposition to ICD therapy and a more active lifestyle.

Migliore et al. (78) showed an elevated rate of complications in young patients with channelopathies and cardiomyopathies with a 9% of patients having inappropriate shocks and 21% device-related complications (need for reoperation, infection, venous occlusion, lead failure, ICD storms). Annual rate of device or lead related complications was 2–4%.

Similarly, in the study by Maron et al. (27) ICD-related complications in over 200 HCM patients occurred in 41% with inappropriate shocks and lead malfunction being the most frequently observed. Notably, most patients received the ICD for primary prevention.

Another important issue that should be taken into account when dealing with ICDs in pediatric age is that in very small children ICD implantation and programming is challenging due to small body size, elevated heart rates and a rapid increase in weight and height. Therefore, for this small subgroup of patients, non-transvenous ICD (NT-ICD) are often the only option. Different techniques can be used (i.e., abdominal device/subcutaneous shock coil or subcardiac device and pleural shock coil); however, they are all burdened by a high incidence of surgical revision and complications (79).

**Figure 3:**
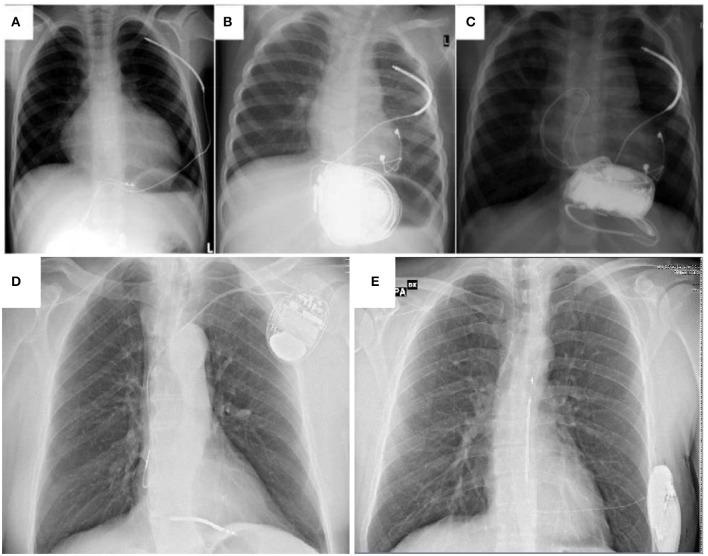
A comparison between non-transvenous ICD **(A–C)** transvenous ICD **(D)** and subcutaneous ICD **(E)** in children and young patients affected by cardiomyopathies. **(A)** extracardiac ICD system with abdominal device and subcutaneous shock coil. **(B)** extracardiac ICD system with abdominal device and pleural shock coil. **(C)** Same as in **(B)**, an additional set of epicardial pace-sense leads was sutured to the right ventricle and the device was fixed in a subcardiac position **(A–C)** from Müller et al. (79) Transvenous and non-transvenous implantable cardioverter-defibrillators in children, adolescents, and adults with congenital heart disease: who is at risk for appropriate and inappropriate shocks? Europace (2018) 0, 1–8. With permission).

To overcome many of the complications described in T- and NT-ICDs, the subcutaneous ICD (S-ICD) has been developed (80); however due to its size there are only few data supporting the use of such a device in children weighting <25–30 Kg (81), in whom the new intermuscular technique along with the two-incision technique may overcome some of the surgical challenges (33) (Figure 3).

The S-ICD is a new technology which has been studied for adult patients without an indication for brady and antitachycardia (ATP) or biventricular pacing (Figure 3). The safety and efficacy of S-ICD were analyzed in the EFFORTLESS and IDE registries (34), with a 1-year rate of complications and inappropriate shocks, respectively, 8,4 and 8,1%. Cardiomyopathies and arrhythmic disorders were well-represented in these studies. HCM patients were evaluated extensively and the device efficacy was proved; defibrillation failure was mostly due to obesity (82, 83). The recent improvements in algorithms to discriminate arrhythmias and minimize T-wave oversensing have further reduced inappropriate shocks in this population.

DCM patients may also be good candidates for S-ICD, unless they have an indication to resynchronization therapy, since the need for pacing is generally low and the role of ATP is unclear (84). On the contrary, ATP has appeared very useful in terminating VTs in AC patients. Nevertheless, young patients tend to present with VF and S-ICD may be indicated in the absence of previous SVTs.

When considering S-ICD, screening selection should be taken into account. Predictors of screening failure in younger patients are longer QRS duration, longer QTc and a lower R/T ratio (85), similar to what observed in adults (86, 87).

Children are underrepresented in the largest studies on S-ICD (80), however few small studies outlined the efficacy and safety of S-ICD in children affected by cardiomyopathies.

Pettit et al. (88) analyzed 17 children with S-ICD (9 pts) and T-ICD (8 pts) and showed a survival benefit and lower complication rate with S-ICD.

Griksaitis et al. (89) reported the UK experience on 23 patients (median age 13 and weight 41 kg) with conventional (13 pts), non-transvenous (7 pts), and totally subcutaneous ICD (3 pts). They showed that in childhood and post-pubertal growth the completely subcutaneous system is the best option unless pacing is needed since it is effective in preserving the vascular system and in avoiding lead related problems.

In 2018 Silvetti et al. (90) presented the Italian experience on 15 patients (mean age 15 years, BMI 22,6 ± 3,4) all implanted with S-ICD, mainly for primary prevention (93%). This is the largest population of patients <18 years so far analyzed.

ICD implantation resulted to be safe and effective with a low rate of appropriate shocks, even though 27% of patients had device related complications that required surgical intervention, with a higher risk in those with BMI <20 and in those in whom the 3-incision technique was performed (90).

Finally, for patients at high risk of SCD but not meeting definite indications for permanent ICD, the wearable cardioverter-defibrillator has been proposed as a short-term solution (91, 92). However, there may be some limitations in children according to anthropometric parameters. Indeed, pediatric patients must have a minimum chest circumference of 66 centimeters and a weight of 18 kilograms or greater to be candidate for the LifeVest.

## Conclusions

Data on risk stratification in the pediatric population affected by cardiomyopathies are limited and risk evaluation is generally performed following criteria derived from the adult population. As children may be at higher risk of short and long term device complications, registries including big numbers of pediatric patients are warranted to improve risk stratification and management in this age subgroup.

Furthermore, cardiac devices are mainly studied for adults and there is limited economic interest in developing specific devices for the pediatric age due to the small number of children that may benefit from ICD implant. However, the scientific community has the duty to raise such an issue and probably ICDs for pediatric patients should be developed outside the conventional market and designated as Orphan Medical Products.

## Author Contributions

All authors contributed to writing and revision.

### Conflict of Interest

The authors declare that the research was conducted in the absence of any commercial or financial relationships that could be construed as a potential conflict of interest.
